# NRG1 Regulates Proliferation, Migration and Differentiation of Human Limbal Epithelial Stem Cells

**DOI:** 10.3390/cimb45120632

**Published:** 2023-12-14

**Authors:** Bofeng Wang, Huizhen Guo, Zhuo Han, Siqi Wu, Jiafeng Liu, Zesong Lin, Fengjiao An, Jin Zhu, Mingsen Li

**Affiliations:** State Key Laboratory of Ophthalmology, Zhongshan Ophthalmic Center, Sun Yat-sen University, Guangdong Provincial Key Laboratory of Ophthalmology and Visual Science, Guangzhou 510060, China; wangbf6@mail2.sysu.edu.cn (B.W.); hanzh9@mail2.sysu.edu.cn (Z.H.);

**Keywords:** limbal epithelial stem cells, gene expression dynamics, proliferation, differentiation, migration

## Abstract

Limbal epithelial stem/progenitor cells (LESCs) proliferate, migrate and differentiate into mature corneal epithelium cells (CECs) that cover the ocular surface. LESCs play a crucial role in the maintenance and regeneration of the corneal epithelium, and their dysfunction can lead to various corneal diseases. Neuregulin 1 (NRG1) is a member of the epidermal growth factor family that regulates the growth and differentiation of epithelial tissues. Here, we depicted the dynamic transcriptomic profiles during human CEC differentiation, identifying six gene co-expression modules that were specific to different differentiation stages. We found that the expression of *NRG1* was high in human LESCs and decreased dramatically upon differentiation. Knockdown of *NRG1* significantly inhibited LESC proliferation and upregulated the expression of the terminal differentiation marker genes *KRT3*, *KRT12* and *CLU*. In addition, the scratch wound closure assay showed that knockdown of *NRG1* attenuated wound closure of LESCs over 24 h. Together, we dissected the transcriptional regulatory dynamics during CEC differentiation and identified NRG1 as a key regulator that promoted LESC proliferation and migration and maintained the undifferentiated state.

## 1. Introduction

As a protective barrier against external insults, the corneal epithelium consists of stratified layers of corneal epithelial cells (CECs), located on the anterior surface of the eye [1,2]. The corneal epithelium plays an essential role in maintaining corneal transparency and protecting the ocular surface from injury or microbial invasion [3]. The corneal epithelium is continuously regenerated by a population of limbal epithelial stem/progenitor cells (LESCs) located in the basal layer of the limbal epithelium [3,4]. Generation of the corneal epithelium involves proliferation, differentiation, migration and adhesion of LESCs [1,2]. Dysfunction or deficiency of LESCs can lead to disruption of the corneal epithelium and various ocular pathologies, such as dry eye syndrome, infections, corneal ulcers and erosions [5,6]. The essential role of LESCs in maintaining corneal epithelial homeostasis underscores their importance for ocular health and clear vision. However, the molecular mechanisms underlying LESC proliferation and differentiation remain largely unknown.

Neuregulin 1 (NRG1), a member of the epidermal growth factor family, is a membrane glycoprotein that regulates the growth and differentiation of multiple organ systems, including the intestinal epithelium [7,8], neuronal system [9] and skeletal muscle [10]. Diseases such as lung cancer, bipolar disorder and schizophrenia have been reported to be associated with dysfunction of NRG1 [11,12]. A previous study suggested that NRG1 is activated after intestinal tissue damage and promotes intestinal stem cell proliferation and regeneration [7]. A previous study indicated that NRG1 is expressed in the adult human cornea [13]. However, the function of NRG1 in LESCs remains unclear. In this study, we depicted the gene expression dynamics regulating CEC differentiation. We found that NRG1 was expressed in LESCs. Knockdown of *NRG1* significantly inhibited LESC proliferation. In contrast, *NRG1* knockdown activated the expression of CEC marker genes. Furthermore, we found that NRG1 was able to promote LESC migration. Taken together, our findings suggest that NRG1 plays a key role in proliferation, migration and differentiation of LESCs.

## 2. Materials and Methods

### 2.1. Normal Human Limbus Samples

Six normal human limbus tissues were obtained as de-identified surgical specimens from the eyebank of the Zhongshan Ophthalmic Center (Guangzhou, Guangdong, China). This study was conducted in accordance with the Declaration of Helsinki and approved by the Ethics Committee of the Zhongshan Ophthalmic Center of Sun Yat-sen University (approval code: 2023KYPJ126).

### 2.2. Isolation and Culture of Human LESCs

Post-mortem human eyes were washed with cold PBS after immersion in DMEM containing 2% penicillin-streptomycin. The limbal samples were separated and cut into small pieces, then incubated with collagenase IV (17104019, Gibco, Waltham, MA, USA) at 37 °C for 1 h. The digested tissue pieces were seeded onto Matrigel-coated (354230, BD Bioscience, Franklin, TN, USA) polystyrene 12-well plates. Cells were incubated at 37 °C in 5% CO_2_ for 10–14 days. The growth medium was refreshed daily. The culture medium was prepared as follows: DMEM/F12 and DMEM (1:1) with 10% fetal bovine serum (16000-044, Gibco, Waltham, MA, USA), 1% penicillin–streptomycin (15140122, Thermo Fisher Scientific, Waltham, MA, USA), 5 mg/mL insulin (I5500, Sigma-Aldrich, Saint Louis, MO, USA), 10 ng/mL EGF (GF144, Millipore, Billerica, MA, USA), 0.4 ug/mL hydrocortisone (386698, Millipore, Billerica, MA, USA), 10^−10^ M cholera toxin (C8052, Sigma-Aldrich, Saint Louis, MO, USA) and 2nM 3,3′,5-triiodo-Lthyronine (T2877, Sigma-Aldrich, Saint Louis, MO, USA).

### 2.3. Immunofluorescence Staining

Cells were fixed with 4% paraformaldehyde for 10 min at room temperature, followed by incubation with a PBS solution containing 0.3% Triton X-100 and 3% BSA at room temperature for one hour. Cells were then incubated with primary antibodies at 4 °C overnight. After washing three times in PBS, cells were incubated with secondary antibodies for 1 h at room temperature. Nuclei were labeled with Hoechst 33258 dye (Thermo Fisher Scientific) for 15 min at room temperature. All images were generated using a Zeiss LSM 800 microscope (Zeiss, Oberkochen, Germany). The antibodies used for immunofluorescence are as follows: anti-KRT3 (Abcam, Cambridge, UK, #ab68260), anti-PAX6 (Sigma, Saint Louis, MO, USA, #AMAB91372), anti-CLU (Proteintech, Wuhan, China, #12289-1-AP), anti-KRT19 (biolegend, San Diego, CA, USA, #628502), anti-KRT12 (Abcam, Cambridge, UK, #ab124975), Anti-KRT14 (Thermo, Waltham, MA, USA, #MA511599), anti-ALDH3A1 (GeneTex, Irvine, CA, USA, #GTX30042), Anti-KI67 (Cell Signaling Technology, Danvers, MA, USA, #9129S) and Anti-TP63 (Cell Signaling Technology, Danvers, MA, USA, 67825S).

### 2.4. In Vitro CEC Differentiation

When LESCs had grown to 100% confluence in LESC medium, the medium was changed to complete keratinocyte serum-free medium (KSFM, Thermo Fisher Scientific, Waltham, MA, USA) with 1.2 μM calcium chloride for one week. The differentiation medium was refreshed daily.

### 2.5. Cell Proliferation Assay

Cell proliferation was measured using the 5-ethynyl-2′-deoxyuridine (EdU) Cell Proliferation Kit (C0071S, Beyotime Institute of Biotechnology, Shanghai, China). Cells were treated with EdU for two hours, followed by fixation, detergent, permeabilization and EdU staining according to the manufacturer’s instructions. 5,6-carboxyflurescein diacetate succinimidyl ester (CFSE) Cell Proliferation Kit (A001, ABP Bioscience, Rockville, MD, USA) was also used to examine cell proliferation. Cells were labeled with 3 μM CFSE for 20 min and then cultured with LESC medium for three days. The CFSE signal intensity was detected by flow cytometry according to the manufacturer’s protocol.

### 2.6. RNA Isolation and Quantitative Real-Time PCR (qRT-PCR)

Total RNA was isolated using the RNeasy Mini Kit (74106, Qiagen, Hilden, Germany). The cDNA was synthesized by reverse transcription using the PrimeScript RT Master Mix Kit (HRR036A, Takara, Kusatsu, Japan) according to the manufacturer’s instructions. qRT-PCR was performed using the iTaq Universal SYBR Green Supermix Kit (1708880, Bio-Rad Life Science, Hercules, CA, USA) in accordance with the manufacturer’s standard method.

### 2.7. Gene Knockdown

Short hairpin RNAs (shRNAs) targeting *NRG1* were subcloned into the PLKO.1 plasmids. For lentiviral packaging, the target and packaging plasmids were co-transfected into HEK293T cells using Lipofectamine 3000 Reagent (L3000015, Thermo Fisher Scientific, Waltham, MA, USA). Lentivirus stocks were collected for two days and filtered through 0.45 μm filter membranes. Cells were infected with lentiviral stocks in fresh LESC medium containing 8 μg/mL polybrene for 24 h. Positive cells were selected in a medium containing 2 μg/mL puromycin for two days. The scrambled shRNA, not targeting any known gene, was used as a negative control under the same conditions. shRNAs targeting *NRG1* were as follows: 5′-CGTGGAATCAAACGAGATCAT-3′ and 5′-GACAGTGCCTCTGCCAATATC-3′. These two shRNAs were pooled for use in all experiments.

### 2.8. Scratch Wound Closure Assay

Cells were seeded onto six-well plates and grown to a confluent monolayer. Scratches were made with a 200 µL pipette tip. The cells were washed two to three times with PBS to remove cell fragments and detached cells. The wounded areas were photographed using a phase-contrast microscope and quantified using ImageJ software (v1.8.0).

### 2.9. RNA-Seq and Data Analysis

Reverse transcription of the sheared RNAs was conducted using the NEBNext RNA First- and Second-Strand Synthesis Module (New England Biolabs, Ipswich, MA, USA). The cDNA was then end-repaired, A-tailed, adapter-ligated and amplified using the KAPA Library Preparation Kit. (Kapa Biosystems, Wilmington, MA, USA). DNA libraries were sequenced on an Illumina NovaSeq 6000 instrument with a paired-end reading length of 150 reads. STAR software (version 2.6.1) [14] was used to map the trimmed reads to the human hg19 reference genome. The transcripts per kilobase million (TPM) values were calculated using the RSEM package (version 1.3.0) [15]. The clusterProfler package (version 3.18.1) [16] was used to perform Gene Ontology (GO) biological process enrichment analysis, with a *p*-value cut-off of 0.05 and a q-value cut-off of 0.05. The Mfuzz package was used to identify gene co-expression modules [17].

### 2.10. Statistical Analysis

We performed statistical analysis with GraphPad Prism using the Student’s unpaired two-tailed *t*-test. Statistically significant differences were denoted by a *p*-value < 0.05.

## 3. Results

### 3.1. Gene Expression Profiles during CEC Differentiation

**Figure 1:**
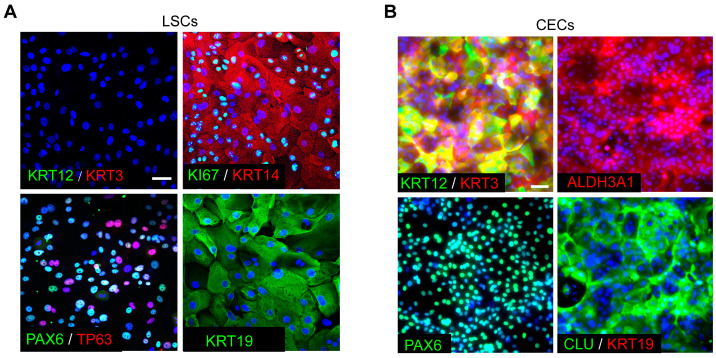
Identification of primary human LESCs and induced CECs. (**A**) Immunofluorescence staining for the indicated genes in primary human LESCs. Scale bar, 100 μm. (**B**) Immunofluorescence staining for the indicated genes in CECs that were differentiated from LESCs in vitro. Scale bar, 100 μm.

**Figure 2:**
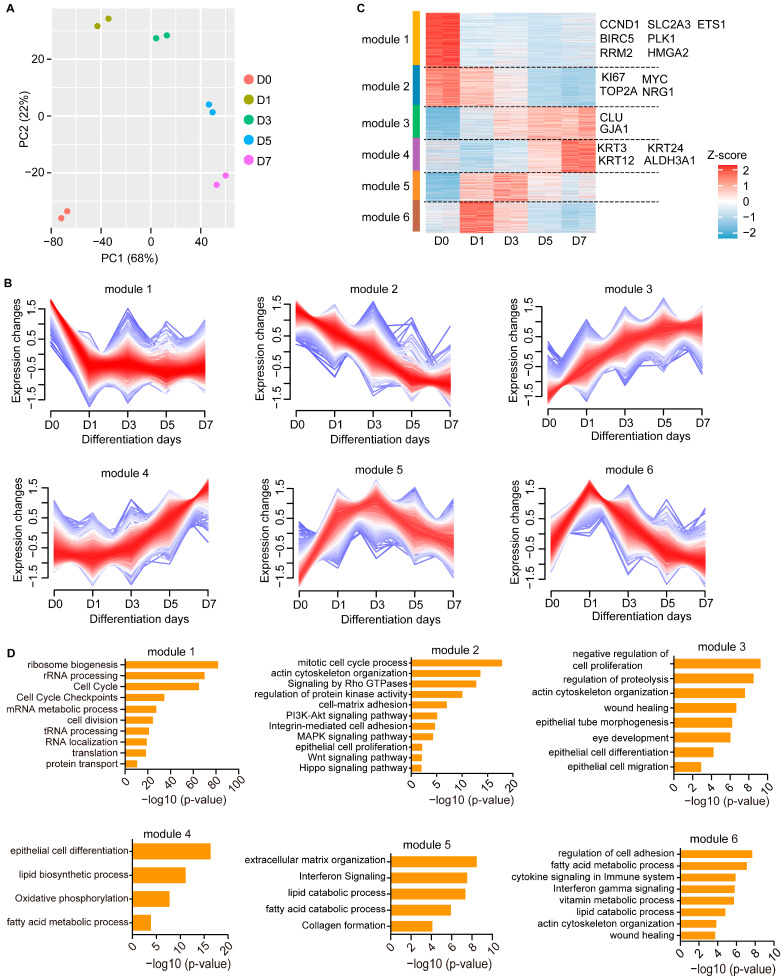
Gene expression dynamics during CEC differentiation. (**A**) PCA for transcriptome data of LESCs (Day 0) and CECs that were differentiated from LESCs for 1, 3, 5 and 7 days. (**B**) Mfuzz time series cluster diagram. (**C**) Heatmap showing gene expression of the Mfuzz modules. (**D**) GO enrichment analysis for each Mfuzz module.

To investigate gene expression profiles during CEC differentiation, we first isolated and expanded primary human LESCs in vitro. These LESCs showed high stemness and proliferative capacity, as evidenced by the expression of the marker genes TP63, KI67, KRT14, KRT19 and PAX6 (Figure 1A). As expected, the CEC markers KRT3 and KRT12 were not expressed in these LESCs (Figure 1A). LESCs were then induced to differentiate into mature CECs using differentiation medium as previously described [18]. The CEC-specific markers KRT3, KRT12, ALDH3A1 and CLU, but not KRT19, were expressed in the induced CECs (Figure 1B). To explore the gene expression dynamics during CEC differentiation, we performed an RNA-Seq assay for five differentiation time points (day 0, day 1, day 3, day 5 and day 7). Principal Component Analysis (PCA) revealed a good separation between the five groups, suggesting that each differentiation stage harbored distinct gene expression patterns (Figure 2A).

### 3.2. Temporal Dynamics of Transcriptional Regulation during CEC Differentiation

To dissect the gene expression changes during CEC differentiation, we performed Mfuzz time-course clustering analysis of these RNA-Seq datasets, identifying six gene co-expression modules (Figure 2B,C). We then performed GO analysis for each module. Expression of genes in module 1 was high only in LESCs and decreased dramatically upon differentiation (Figure 2B). As expected, module 1 was associated with cell cycle, including the LESC proliferation genes *ETS1*, *HMGA2*, *CCND1*, *BRIC5*, *RRM2*, *SLC2A3* and *PLK1* [19] (Figure 2C,D). Module 1 was also involved in ribosome biogenesis, translation, protein transport, the mRNA process and the non-coding RNA process, suggesting higher transcriptional and translational activities in LESCs (Figure 2D). In contrast, the expression of module 2 genes such as *MYC* [20], *KI67* and *TOP2A* [19] progressively decreased during differentiation (Figure 2C). Module 2 included genes important for epithelial ontology and functions, including proliferation, adhesion and actin cytoskeleton organization (Figure 2D). The WNT, Hippo, MAPK and PI3K-AKT signaling pathways, which promote corneal epithelial proliferation [21,22,23,24], were significantly enriched within module 2 (Figure 2D).

Module 3, including the CEC markers *CLU* and *GJA1*, showed continuous up-regulation during differentiation and was involved in migration, differentiation, eye development, wound healing, actin cytoskeleton organization and negative regulation of cell proliferation (Figure 2C,D), recapitulating in vivo processes of LESCs. The terminal differentiation markers *KRT3*, *KRT12*, *KRT24* and *ALDH3A1* were assigned to module 4, which was activated at the late differentiation stage (Figure 2C). Interestingly, both the early (modules 5 and 6) and late (module 4) differentiation genes were enriched for biological processes related to lipid and fatty acid metabolism, indicating the potential importance of lipid metabolism to corneal epithelial differentiation (Figure 2D). The genes associated with extracellular matrix organization, wound healing, cell adhesion and immunity signaling were up-regulated at the early differentiation stage (Figure 2D). Collectively, we depicted the dynamic gene regulatory networks during corneal epithelial differentiation.

### 3.3. NRG1 Influences LESC Proliferation and Differentiation

**Figure 3:**
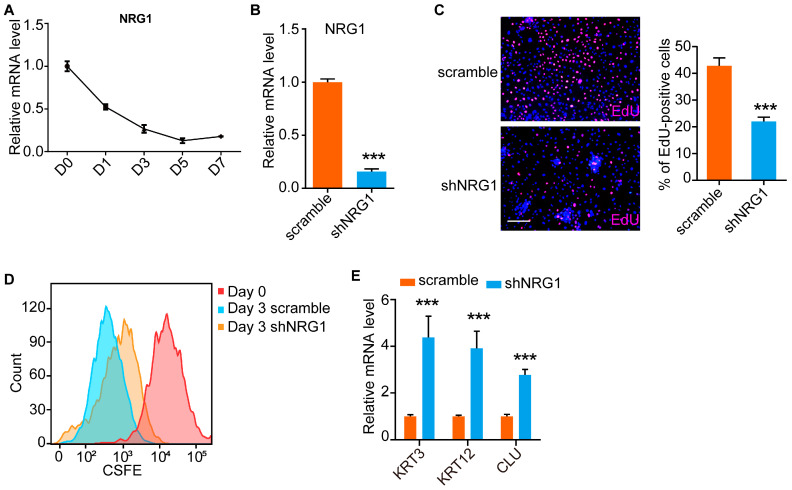
NRG1 influences proliferation and differentiation of LESCs. (**A**) qRT-PCR analysis of the expression level of *NRG1* at the indicated differentiation time points. Data are presented as mean ± SE (n = 3). (**B**) qRT-PCR analysis of the knockdown efficiency of *NRG1*. Data are presented as mean ± SE (n = 3, *** *p* < 0.001). (**C**) EdU staining and quantification analysis after *NRG1* knockdown in LESCs. Scale bar, 200 μm. Data are presented as mean ± SE (n = 3, *** *p* < 0.001). (**D**) CFSE signals in the indicated groups were detected by flow cytometry analysis. Day 0 represents the initial CFSE fluorescence intensity. The CFSE fluorescence intensities in the scramble and shNRG1 groups were quantified on day 3 after CFSE labeling. (**E**) qRT-PCR analysis of the expression of *KRT3*, *KRT12* and *CLU* in shNRG1- and scrambled shRNA-treated LESCs. Data are presented as mean ± SE (n = 3, *** *p* < 0.001).

To reveal the key genes that maintain LESC function, we focused on *NRG1* (Figure 2C), which is module 2-specific and is involved in intestinal stem cell proliferation and maintenance of the stem cell identity [7]. Consistent with the RNA-Seq data, qRT-PCR analysis showed that the expression of *NRG1* was significantly decreased upon differentiation (Figure 3A). We then knocked down the expression of *NRG1* using shRNAs (Figure 3B). EdU staining and CFSE labeling were performed after *NRG1* knockdown. We found that the number of EdU-positive cells was significantly reduced in *NRG1*-depleted LESCs compared to scrambled shRNA-treated LESCs (Figure 3C). Similarly, CFSE labeling experiments showed that *NRG1*-depleted LESCs divided more slowly than did cells in the control group (Figure 3D). In addition, the CEC markers *KRT3*, *KRT12* and *CLU* were activated upon *NRG1* knockdown (Figure 3E). These results suggested that *NRG1* was able to maintain LESCs in a proliferative and undifferentiated state.

### 3.4. NRG1 Promotes LESC Migration

**Figure 4:**
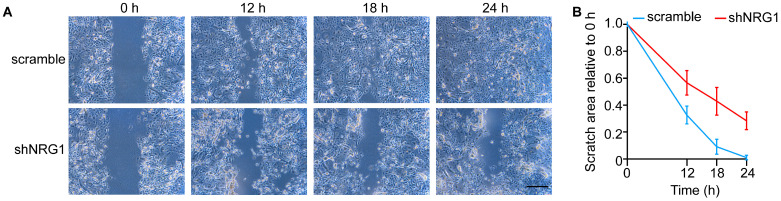
NRG1 promotes LESC migration. (**A**,**B**) Wound-healing assays for shNRG1- and scrambled shRNA-treated LESCs. Representative images (**A**) and quantifications (**B**) of healing areas at the indicated time points. Scale bar, 500 μm. Data are presented as mean ± SE (n = 3).

To investigate the role of *NRG1* in LESC migration, we performed a scratch wound closure assay. We found that *NRG1*-depleted LESCs required a longer time to repair wounded areas (Figure 4A,B), indicating that *NRG1* knockdown inhibited LESC migration. Taken together, these results suggest that NRG1 promoted LESC proliferation and migration and inhibited CEC differentiation.

## 4. Discussion

Corneal epithelial differentiation is a complex process involving the proliferation, migration and differentiation of LESCs [2,4,25]. During this process, the LESCs located at the limbus proliferate, migrate and differentiate to form the multilayered corneal epithelium that covers the corneal surface [25]. LESCs play a crucial role in maintaining the homeostasis of the corneal epithelium by continuously replenishing its cell population. Numerous factors, such as environmental stress, trauma, infection and inflammation, often lead to LESC deficiency, resulting in corneal epithelial disorder and visual impairment [3,5]. Understanding the molecular mechanism underlying the function and identity of LESCs provides clues for the development of innovative therapeutic strategies for corneal diseases and injuries.

An intriguing biological property of LESCs is their proliferation and stemness, which are governed by a set of well-known transcriptional regulators, including TP63 [26], CEBPD [27], KLF7 [28,29] and MYC [20]. Our previous study also showed that ETS1 and HMGA2 are required for LESC proliferation [18]. In this work, we dissected the gene expression changes during CEC differentiation. The identification of LESC-specific gene sets, including many well-established key regulators, implied their potential role in maintaining LESC proliferation and stemness. Interestingly, we found that the genes related to ribosome biogenesis, translation, protein transport and the mRNA process were expressed at higher levels in LESCs than in CECs. The balance between proliferation and differentiation is important for corneal homeostasis. We then identified the gene expression modules specific to the early, mid and late stages of CEC differentiation. We showed that lipid and fatty acid metabolism was specific for CEC differentiation.

LESC function is tightly regulated by various signaling pathways, including the WNT [30,31], NOTCH [32,33,34], TGF-β [30] and BMPs [35,36] signaling pathways. We showed that the WNT, MAPK and PI3K-AKT signaling pathways, which promote LESC proliferation, were specifically expressed in LESCs. NRG1 is a direct ligand for ERBB3 and ERBB4 tyrosine kinase receptors [37]. Here, we found that NRG1 was specifically expressed in LESCs and was able to promote LESC proliferation. Cell cycle withdrawal is important for proper stem cell differentiation. Knockdown of *NRG1* inhibited LESC proliferation and promoted CEC differentiation. Therefore, we identified NRG1 as a key regulator that maintains LESC proliferation and an undifferentiated state. Previous studies suggested that NRG1 can promote wound healing [7,38]. We also found that NRG1 was required for LESC migration. Knockdown of *NRG1* attenuated wound closure of LESCs over 24 h. However, in order to further prove this conclusion, it should be confirmed in the future that the addition of NRG1 protein, both in vivo and in vitro, can accelerate would healing of the corneal epithelium.

## Data Availability

The data presented in this study are available on request from the corresponding author.
